# Amino Functionalized Micro-Mesoporous Hybrid Particles for the Sustained Release of the Antiretroviral Drug Tenofovir

**DOI:** 10.3390/ma13163494

**Published:** 2020-08-07

**Authors:** Araceli Martin-Illana, Raul Cazorla-Luna, Fernando Notario-Pérez, Roberto Ruiz-Caro, Luis Miguel Bedoya, Maria Dolores Veiga-Ochoa, Juan Rubio, Aitana Tamayo

**Affiliations:** 1 Department of Pharmaceutics and Food Technology, Faculty of Pharmacy, Universidad Complutense de Madrid, Plaza Ramón y Cajal s.n, 28007 Madrid, Spain; aracelimartin@ucm.es (A.M.-I.); racazorl@ucm.es (R.C.-L.); fnotar01@ucm.es (F.N.-P.); rruizcar@ucm.es (R.R.-C.); mdveiga@ucm.es (M.D.V.-O.); 2 Department of Pharmacology, Pharmacognosy and Botany, Faculty of Pharmacy, Universidad Complutense de Madrid, Plaza Ramón y Cajal s.n, 28007 Madrid, Spain; lmbedoya@ucm.es; 3 Institute of Ceramics and Glass, CSIC, Kelsen 5, 28049 Madrid, Spain; jrubio@icv.csic.es

**Keywords:** HIV, smart material, hybrid, sol-gel chemistry, drug release

## Abstract

The sustained release of an antiretroviral agent to women mucosa has been proved as an excellent strategy to reduce the sexual transmission of HIV. Hybrid micro-mesoporous particles have been synthesized and functionalized with a silane coupling agent followed by loading the antiretroviral tenofovir. It has been observed that the disposition of the silane molecule on the surface of the particles determines the interaction mechanism with the antiretroviral molecule loaded independently on the surface area of the particles. In this sense, available and free amino groups are required to achieve a smart pH-responsive material, a condition that is only achieved in those materials containing a silane chemisorbed monolayer. Moreover, the modulation of the release kinetics attributed to the presence of the silane monolayer covering the mesopores has been confirmed by fitting the releasing curves to the first order and Weibull models. The developed micro-mesoporous particles have been demonstrated to be excellent smart-release vehicles for antiviral agents and can be safely used in polymer mucoadhesive vaginal gels.

## 1. Introduction

The vast majority of new HIV infections are acquired via the genital and rectal mucosa, with an increased risk of infection in women compared to men. The research in developing microbicides for prevention of mucosal HIV transmission has been focused on using antiretroviral agents in various formulations and dosing strategies. Among the strategies for a sustained release of the antiviral or antiretroviral drugs it stands out the controlled drug delivery systems based on inert materials with high porosity to load the maximum amount of drug. These systems are formed by a support material, organic, inorganic or hybrid where the drug is loaded. Among the multiple possibilities of the porous organic materials, drug delivery systems based on polypropylene, polystyrene, poly(α, β-L Malic acid), poly(ethylene glycol)-block-poly (-caprolactone), ethyl cellulose, etc., can be found in the literature [1,2]. Within the inorganic drug delivery systems, it stands out the mesoporous silica, which can exhibit different kind of interaction with organic molecules to develop biopharmaceutical functions [3,4] SBA-15 and MCM-41 silica are probably the inorganic materials more studied as drug carriers [5,6]. Microporous calcium silicates and carbonates [7,8], magnesium aluminosilicate [7], bioactive glasses and ceramics [9] or titanium oxide [10] have also been explored. These porous materials can be synthesized with high surface area, high pore volume and selected pore size as well as hierarchical pore structure, tuneable hydrophilic-hydrophobic character, thus offering the possibility of bulk or surface modification [11].

In most of the above referred systems, the drug molecules are entrapped inside the pores of the material or physically mixed. Several factors affect the drug release profiles, many of them related to the drug adsorption mechanism. Pore diameter and pore volume determine whether the drug can penetrate into the pore channels or not, the available surface to stablish the drug-host interactions, channel tortuosity and permeability are the main factors affecting the drug loading and releasing capabilities [5]. A common strategy to enhance the adsorption and desorption capabilities of the materials used as a drug carriers is to functionalize the porous surface of the materials, either by the conventional grafting method with coupling agents or through the addition of diverse functionalities to modulate the hydrophilic or hydrophobic properties of the substrate [6,12].

The weak interaction of the inorganic matrixes with the organic (drug) molecules has driven to focus the attention on hybrid materials where the synergistic combination of both inorganic and organic components makes them suitable for a wide range of medical applications. The flexible sol-gel procedure is an excellent route to prepare inorganic-organic hybrid materials where homogenous precursor solutions yield either non porous or porous gels in a wide size range. Silicon-based organic-inorganic hybrid and ceramics materials obtained by sol–gel process have been extensively studied over the past years due to their promising biomedical applications [13,14,15]. The application of the silicon polymers is not new in the field of the biotechnology since we can find several examples such as catheters [16], medical tubes [17] or contact lenses [18]. The selective modification of their surface characteristics allows to provide specific interaction of the bio-interfaces with living body tissues and, more recently, in microfluidics [19].

Among the drugs used in vaginal microbicide development, reverse transcriptase inhibitors act on viral enzyme reverse transcriptase, being the tenofovir (TFV) one of the most promising candidates since it is a safe molecule and possess a long half-life [20]. In a smart drug delivery system, the drug release starts with the diffusion of the drug molecule through the smaller pores where the drug must interact with the surrounding media and afterwards, diffusion through the material pores to reach the bulk dissolution medium takes place. When the system is composed of porous inorganic particles mixed in a non- soluble matrix the diffusion of the drug molecules is highly restricted and the drug delivery may be sustained for long periods of time.

The purpose of this work is to develop highly porous materials acting as antiviral drug carriers from the partial pyrolysis of hybrid sol-gel derived precursors. Organic-inorganic hybrids were subjected to a heat treatment at temperature below the mineralization reaction (where the organic component of the hybrid has almost disappeared). Our purpose is to modify the surface characteristic of the materials through direct functionalization with a silane coupling agent. In the modified materials, the antiviral TFV drug has being loaded, and it turned out that the amount of drug adsorbed on the surface of the material is not dependent on the specific surface nor the functionalization but on the chemisorption of the silane instead. After loading the maximum amount of drug molecule in each of the different materials, the amount of drug released in simulated vaginal fluid was determined.

These hybrid particles are intended to take part of a mucoadhesive vaginal composite, as described by Veiga-Ochoa et al. [21,22,23,24,25,26]. After selecting the appropriate carrier, it must be included into the vaginal formulation for in-vivo tests, processability and comfortability assays.

## 2. Materials and Methods

### 2.1. Synthesis and Functionalization of the Porous Hybrid Particles. Characterization Methods

Triethoxysilane (TrEOS, ABCR, Karlsruhe, Germany) was mixed with polydimethyl siloxane (PDMS, ABCR, Karlsruhe, Germany) in a weight ratio of 70:30 and stirred in isopropanol (iPROH, Sigma Aldrich, St. Louis, MO, USA, 98%) in a vessel at room temperature. The solution was put into a reactor thermostatized at 70 °C and under reflux and subsequently, a solution containing iPrOH, H_2_O and Hydrochloric acid (HCl, Fluka, Charlotte, NC, USA, 32%) was incorporated. The molar ratio of the different components was 4.5:3:0.05:1 (iPrOH:H2O:HCl:Triethoxysilane). The solution was left reacting for 1 h at 70 °C with constant stirring. The obtained sol was poured to a plastic container and after 30 min, a solution of NH_4_OH 1 M was slowly added (caution, vapor gases emerged). The containers were maintained closed for 1 week. Periodically, the gases were released to avoid overpressure. The gels were first dried at 50 °C and 120 °C until constant weight and they were pyrolyzed under a constant N_2_ flow of 180 mL min^−1^ at temperatures ranging from 500 to 800 °C. Heating and cooling rate was fixed to 8 °C min^−1^ and the materials were maintained at the maximum temperature for 2 h.

**Scheme 1:**
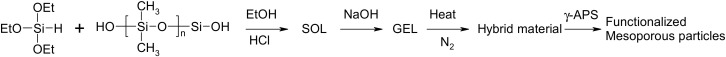
General scheme of the synthetic procedure.

The porous surface of the obtained hybrid materials was modified by grafting -aminopropyl trimethoxy silane (APS, Sigma Aldrich, St. Louis, MO, USA, 99%) molecules in aqueous medium. APS was hydrolysed for 1 h in H_2_O at 25 °C and afterwards 1 g of the hybrid particles was added. Coupling the silane molecules takes 30 min under vigorous stirring and then the surface modified particles were filtered, dried at 50 and 120 °C. We have used APS/H_2_O w/w ratios of 0.3%, 0.5%, 1%, 2% and 3%. In the following scheme (Scheme 1), we describe the principal stages of the synthetic procedure and the subsequent functionalization of the obtained particles.

Attenuated Total Reflectance (ATR) spectra were obtained with a Perkin-Elmer spectrophotometer instrument equipped with a MIRacle^TM^ accessory designed for ATR measurements (Perkin-Elmer, Waltham, MA, USA). Thermal analysis was carried out in a TA Q600 thermobalance fed with a constant air flow of 100 mL/min The samples were placed into platinum crucibles and the mass change and heat flow was continuously recorded while heating at 10 °C/min. The specific surface areas (SSA) calculated by the BET equation [27], pore size distributions (PSD) with the corresponding calculations of the mesopore surface areas (MSA) and mesopore volumes (MPV) were obtained by the Barrett, Joyner and Halenda (BJH) procedure [28] and obtained from the nitrogen adsorption isotherms at 77 K (Tristar 3000, Micromeritics, Norcross, GA, USA). Microscope images were collected in a Field-Emission Scanning Electron Microscope (FE-SEM) HITACHI S-4700. Gold was sputtered on the surface of the particles to provide the electronic conductivity.

### 2.2. Cytotoxicity Evaluation

In these experiments we used three human cell lines: The MT-2 lymphoblastic cell line, the macrophage-monocyte derived cell line, THP-1 (ATCC® TIB-202) and an uterus/endometrium epithelial cell line, HEC-1-A. The cell lines were cultured at 37 °C in a humidified atmosphere of 5% CO_2_ in RPMI 1640 medium supplemented with 10% (v/v) fetal bovine serum, 2 mM L-glutamine and 50 mg mL^−1^ streptomycin (all Whittaker M.A. Bio-Products, Walkerville, MA, USA). To detach HEC-1-A cells, the medium was removed and the flask was thoroughly rinsed with 1 to 2 mL of Trypsin 0.25% -EDTA 0.03% solution. The medium was replaced every three days after cell centrifugation at 1500 rpm for 5 min.

All the cell lines were incubated in 96-well plates at a density of 10 × 10^5^ cells per well (MT-2 and THP-1) and 2 × 10^4^ (HEC-1-A) in complete medium. To assess the cytotoxic effect, the cells were exposed to fresh medium containing different concentrations of suspensions of 5J, 6J, 7J or 8J or the same concentration of PBS 1× as control. A standard method was followed to suspend the materials in PBS 1× [29]. The experiments were performed in triplicates and the culture was maintained at 37 °C and 5% CO_2_ humidified atmosphere for 48 h incubation. After incubation, the media was removed from the cell cultures and 50 µL of CellTiter Glo (Promega, Madison, WI, USA) reactive was then added to each well of the plate. Relative luminescence Units (RLUs) were measured in a luminometer (Sirius, Berthold Detection Systems). Cytotoxic concentrations 50 values (CC_50_ values) were calculated using GraphPad Prism Software (San Diego, CA, USA).

### 2.3. Drug Loading and Release Experiments

The porous particles were immersed in an aqueous solution of tenofovir (TFV, Carbosynth Limited, Berkshire, UK) of a known concentration. They were maintained in a bath thermostated at 37 °C under vigorous stirring for 180 min or until the maximum loading capacity of the material was achieved. The amount of TFV loaded was determined by UV-vis spectroscopy (Labmda 25, Perkin-Elmer, Waltham, MA, USA) at the wavelength of 320 nm corresponding to the maximum absorption of TFV. After selecting the optimal time to achieve the maximum loading, the particles were immersed in Simulated Vaginal Fluid (SVF) [30] thermostated at 37 °C under continuous shaking. The SVF (pH 4.2) was prepared according to the established method [31]. During loading, the shaking speed was fixed to 45 opm whereas releasing experiments were performed under steady conditions at 15 opm. Drug release studies were carried out at 37 °C in SVF under stirring at 200 rpm in closed containers. 100 mg of loaded particles were immersed in 100 mL SFV and 4 mL aliquots of the solution were taken at certain time intervals. Then, 4 mL of fresh SVF was added to the solution. As in the drug loading experiments, the amount of TFV in solution was determined by UV-vis spectroscopy.

## 3. Results

### 3.1. Characterization of the Functionalized Porous Hybrid Particles

**Figure 1:**
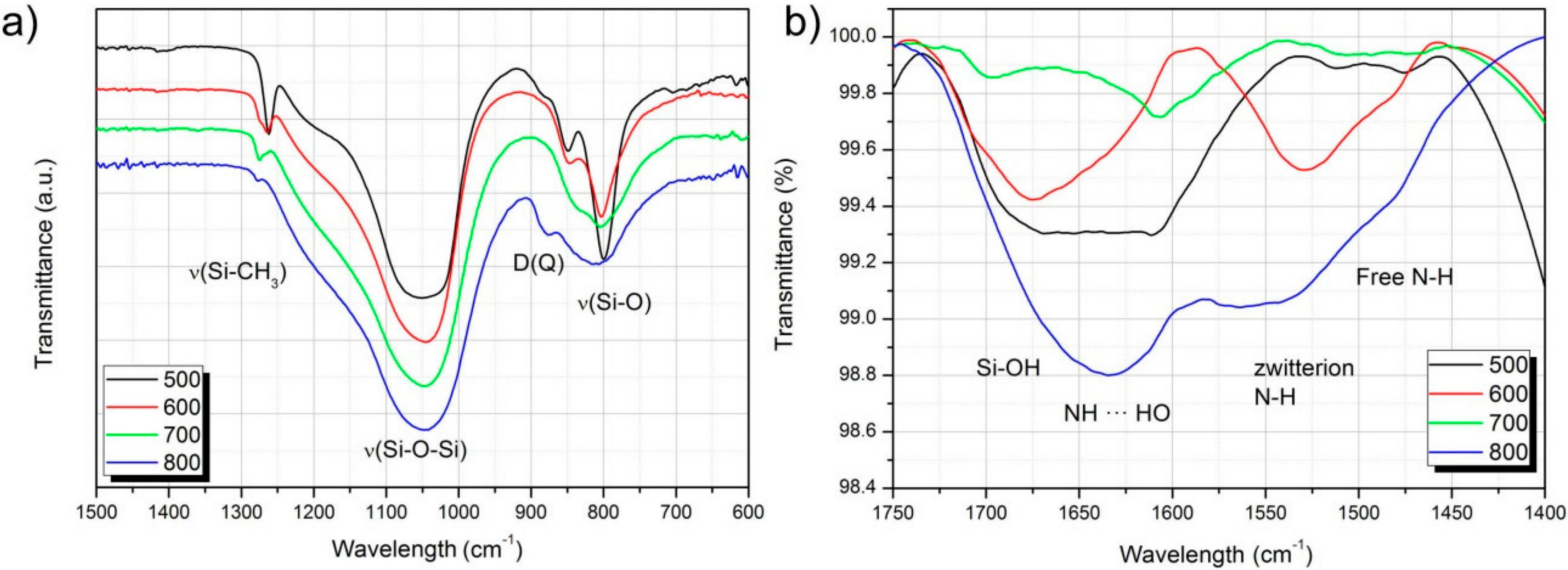
(**a**) FTIR spectra of the materials pyrolyzed at different temperatures and (**b**) detail of the FTIR-ATR spectra of the materials pyrolyzed at different temperatures and functionalized with 1% silane in solution.

Fourier transform infrared (FTIR) spectroscopy was used for the structural characterization of the porous hybrid particles as well as for the determination of the interaction between the silane coupling agent and the surface of the particle. Figure 1a presents the FTIR spectra in the transmission mode of the materials pyrolyzed at different temperatures. As observed, the characteristic symmetric bending of CH_3_ in the PDMS appearing at 1264 cm^−1^ diminishes its relative intensity as the pyrolysis temperature increases and experiments a slight blue shift due to the decomposition into =CH_2_ terminal units while the glass conversion. The most intense band of the spectra occurs due to the contribution of the longitudinal and transverse optical vibration modes of the siloxane bonds (Si–O–Si) in the hybrid network. The center of the band, appearing at 1080 cm^−1^, corresponds to the vibration of the network more similar to pure silica while the LO mode of the hybrid network is slightly displaced to higher frequencies when organic character increases [32,33]. The observed red shift due to the inorganic conversion occurs together with an increase of the wavelength span, a broadness which is more evident in the band attributed to the symmetric tension of the Si−O bonds (band centered at 800 cm^−1^).

Babboneau et al. [34] described the transformation of the weak band corresponding to D units in PDMS polymers to a stronger band shifted to 850 cm^−1^ when D and Q units are present in the copolymer. As occurred with the band located at 1264 cm^−1^, the pyrolysis temperature raise induces the progressive disappearance of the PDMS-related band.

Due to the inherent adsorption of H_2_O in the KBr diluted samples, FTIR-ATR spectroscopy with no KBR dilution was performed to identify the different interactions between the grafted γ-APS molecules and the surface of the hybrid materials. The presence in each silane molecule of three hydroxyl groups (Si–OH) and an amine terminated organic chain with the NH_2_ group at the end (–CH_2_–CH_2_–CH_2_–NH_2_), different interactions are feasible. Figure 1b shows the infrared spectra of the different materials containing 1% γ-APS in the spectral range 1400–1750 cm^−1^ where it is clearly observed that, depending on the pyrolysis temperature, the interaction of the coupling agent with the surface of the hybrid occur in a similar fashion. This interaction does not exclusively depend on the pyrolysis temperature (i.e., the content of organic phase) but also on the amount of γ-APS. Supplementary Material Figure S1 collects the FTIR spectra of all the materials with the different concentrations of γ-APS incorporated.

Free amino groups are shown at around 1520 cm^−1^ whereas the band located at 1590 cm^−1^ wavelengths appears due to hydrogen interactions (zwitterionic forms between the –NH_2_ and Si–OH) with the surface of the material [35,36]. Weak interactions with other amino or silanol groups of adjacent APS molecules shift the zwitterionic band to higher wavelength. Here, we observe that, for the same silane coupling agent concentration, at 500 °C the preferred interactions are –SiOH–H_2_N– with no interactions between the amino groups of the silane molecules by increasing the temperature, the NH_2_–H_2_N acquire more relevance.

**Figure 2:**
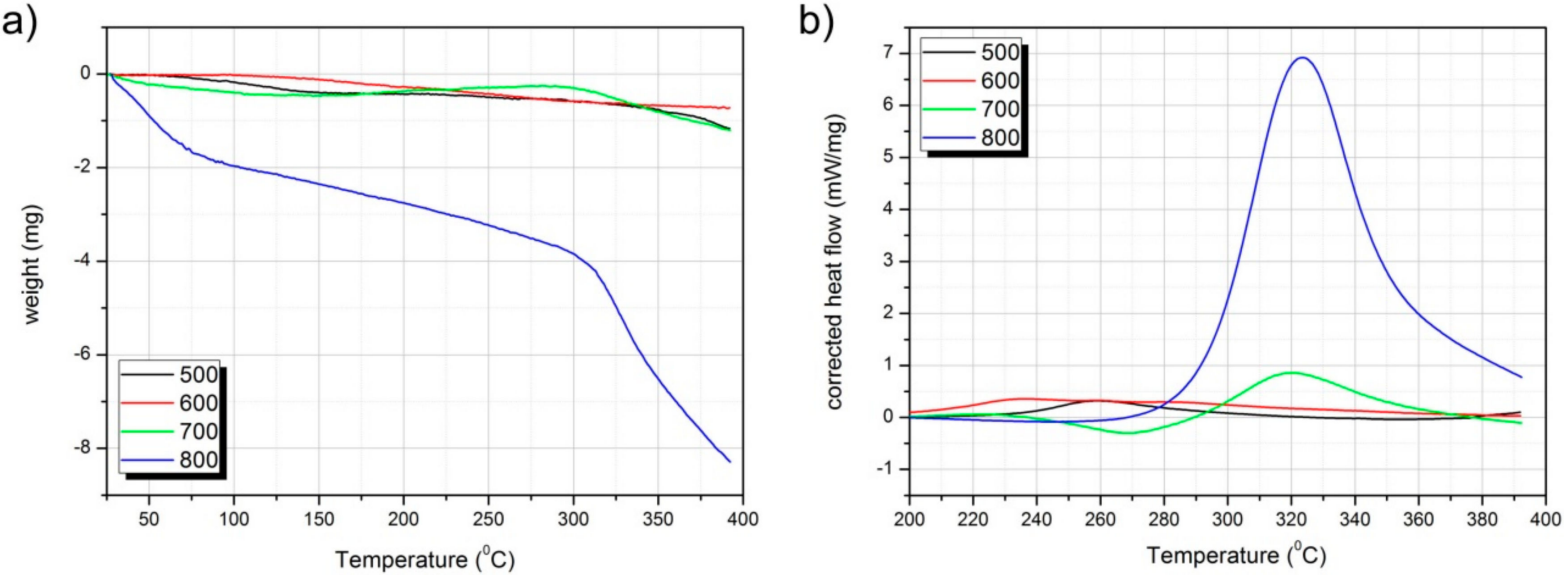
(**a**) Thermogravimetric and (**b**) differential scanning calorimetry curves of the materials pyrolyzed at different temperatures and containing 1% γ-APS in solution (the contribution to the non-functionalized substrate has been subtracted to the thermogrammes).

The relative intensity of these spectra cannot be taken as an evaluation of the amount of γ-APS adsorbed on the surface of the materials because of the inherent characteristics of the ATR measurements (different incidence angle in particles and porosity). We have used the thermogravimetric analysis (Figure 2a) for the calculation of the amount of γ-APS adsorbed on the surface of the material (Supplementary Material Figure S2). As observed in Figure 2a, the material which has incorporated the maximum amount of the silane coupling agent is the one pyrolyzed at 800 °C since it shows the maximum weight loss with the increase of the temperature. In this sample, it is noticed a first weight decrease that does not correspond to the desorption of the silane molecule but H_2_O adsorbed on the surface. The differential scanning calorimetry curves (Figure 2b) suggest that the adsorption mechanism on the surface of the material is different dependent upon the pyrolysis temperature. At 500–600 °C pyrolysis, the preferential adsorption mechanism is the physisorption of the molecules while in the samples pyrolyzed at 700 and 800 °C the coupling agents are chemisorbed. The formation of a functional silane monolayer on the surface of materials is one of the factors critical to the adhesion. This minimizes the formation of physisorbed silanes, i.e., a weakly bonded multi-layer coating that affects the functionality of the material in an aggressive environment.

The analysis of the DSC curves of the different materials (Supplementary Material Figure S3) show that the dependence of the adsorption mechanism is not exclusive on the pyrolysis temperature but on the amount of silane in solution as well. In the samples pyrolyzed at low temperature (500 and 600 °C), the coupling agent is firstly physiosorbed and, by increasing the amount of silane in solution, it becomes to be chemically adsorbed. The opposite happens in the materials pyrolyzed at the highest temperatures. The silane is chemically adsorbed on the surface of the material until the formation of the monolayer and afterwards, the physisorption mechanisms dominates the functionalization.

The decrease of the specific surface area (SSA) is another evidence of the efficiency of the functionalization. Except for the samples pyrolyzed at 500 and 600 °C, there is a marked decrease of the SSA with the amount of silane in solution (Table 1). The isotherms (Supplementary Material Figure S4) are of type VI, according to the IUPAC classification [37], characteristic of micro-mesoporous materials. It can be observed two types of hysteresis loops, being the one observed in the high partial pressure range (larger mesopores) of a type H3, indicating slit-shaped pores and, at lower partial pressure (smaller pores), the pores present a cylindrical shaped, as indicated from the H2 hysteresis loop [38].

**Figure 3:**
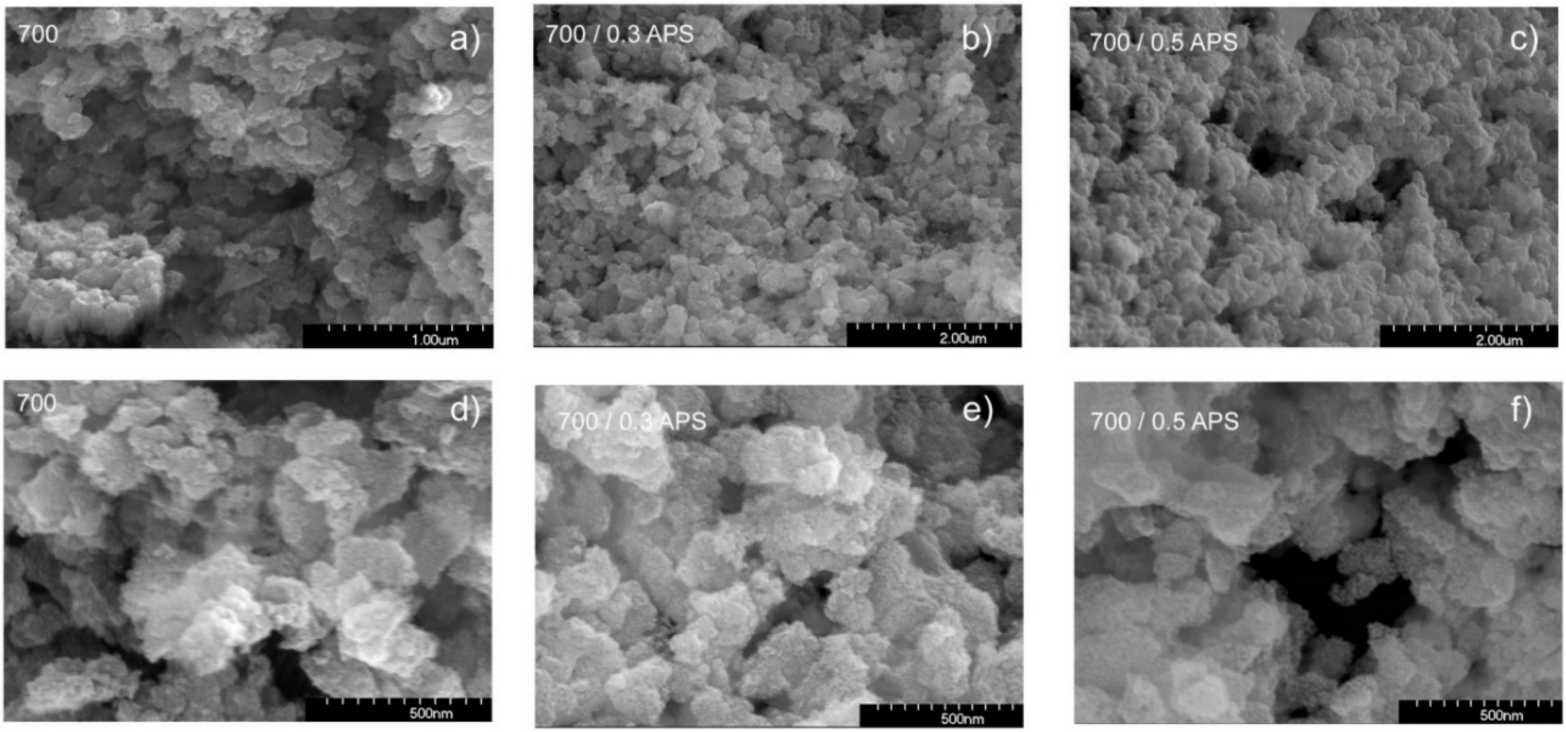
Scanning Electron Microscopy images of the particles pyrolyzed a 700 °C and functionalized with the silane coupling agent γ-APS in solutions containing (**a**) and (**d**) 0% APS, (**b**) and (**e**) 0.3% APS and (**c**) and (**f**) 0.5% APS. Magnification 40 k for images (**a**)–(**c**) and 70 k for images (**d**)–(**f**).

The SEM images demonstrate the porous character of the particles (Figure 3). When the hybrid material is immersed in the solutions containing the silane coupling agent, a slight aggregation of the particles is observed, but in any case they lose their nanostructured character of the surface. As can be observed in the high magnification images, the surface of the particles is completely covered by nanosized aggregates which must be responsible of the appearance of the microporosity.

### 3.2. Toxicity Evaluation

To study the cellular toxicity, the synthesized particles were incubated in culture media at room temperature for 48 h before the assay, to assure that any potential toxic component will be present in the dilutions to be tested. Afterwards, the cell culture was treated with a suspension of base 5 serial dilutions of the more concentrated suspension (1000 µg/mL). The experiments performed in lymphoblastic (MT-2) and in macrophage-monocyte derived cell lines were carried out to evaluate their toxicity on immune cells presents in vaginal or uterus mucosae, and the experiment in a uterus epithelial cell line (HEC-1A) served to evaluate the potential damage on the mucosae integrity.

**Figure 4:**
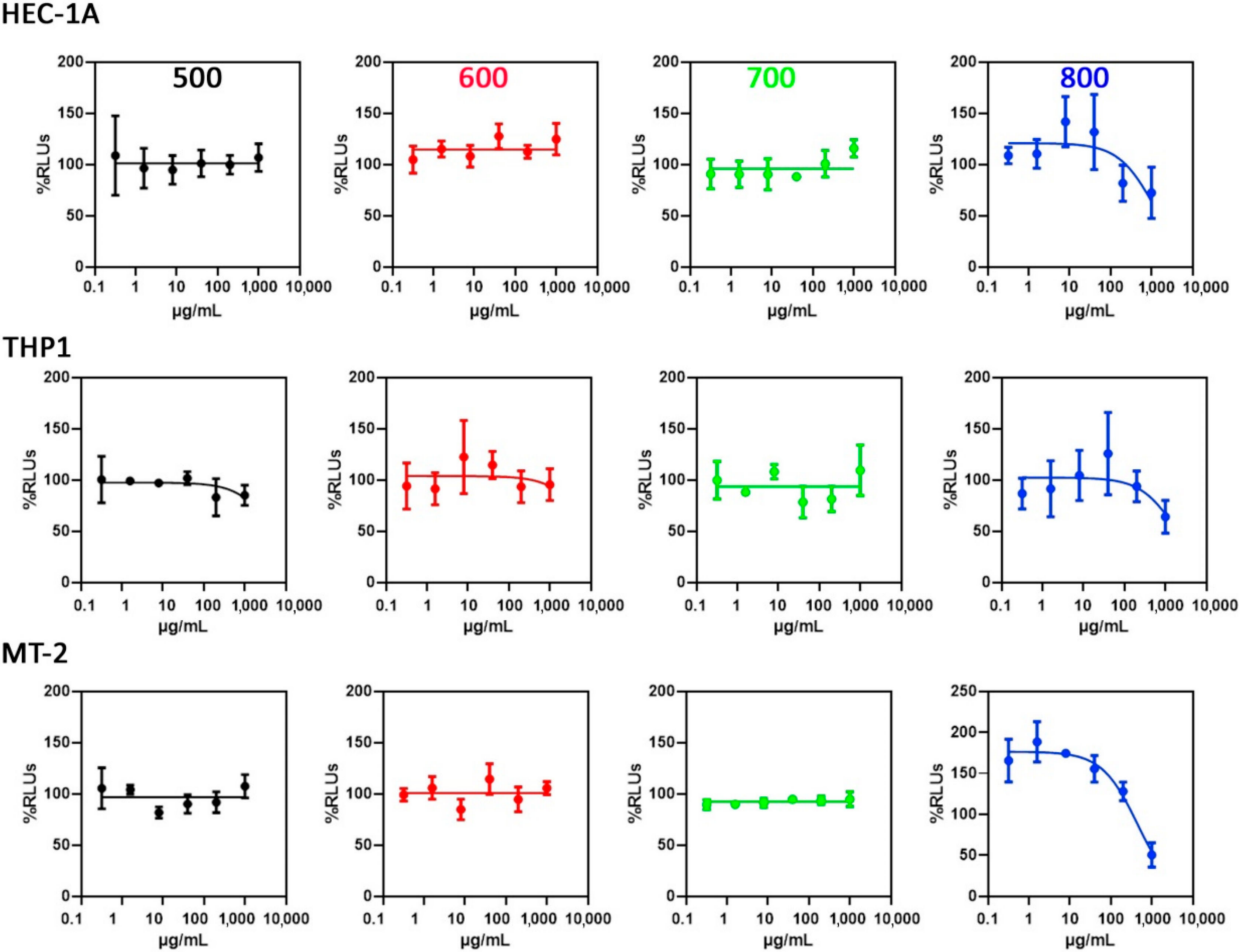
Graphic representation of the cytotoxic evaluation of the particles pyrolyzed at 500, 600, 700 and 800 °C in MT-2, THP-1 and HEC1A cells. Cell viability is expressed as percentage of living cells (%RLUs) as compared to a non-treated control (100%). RLUs: Relative luminescence units.

As shown in Figure 4 and Table 2, the samples pyrolyzed at 700 °C and below are all of them biocompatible in the three cell types, displaying CC_50_ values greater than 1000 µg/mL, the maximum concentration tested. Only the sample pyrolyzed at 800 °C showed cell toxicity in the three cell types, showing a slightly higher toxicity in MT-2 cells, the lymphoblastoid cell line, with a CC_50_ value of 447.5 µg/mL. However, toxic effects on monocyte THP-1 cells and epithelial cells HEC-1A only appeared at concentrations around 1000 µg/mL, displaying a toxic effect only at high concentrations. Since the size and shape of the particles were maintained constant in all the cases, the different surface functionalities of the particles might be the responsible of the observed cytotoxic behavior.

### 3.3. Drug Loading Capacity

**Figure 5:**
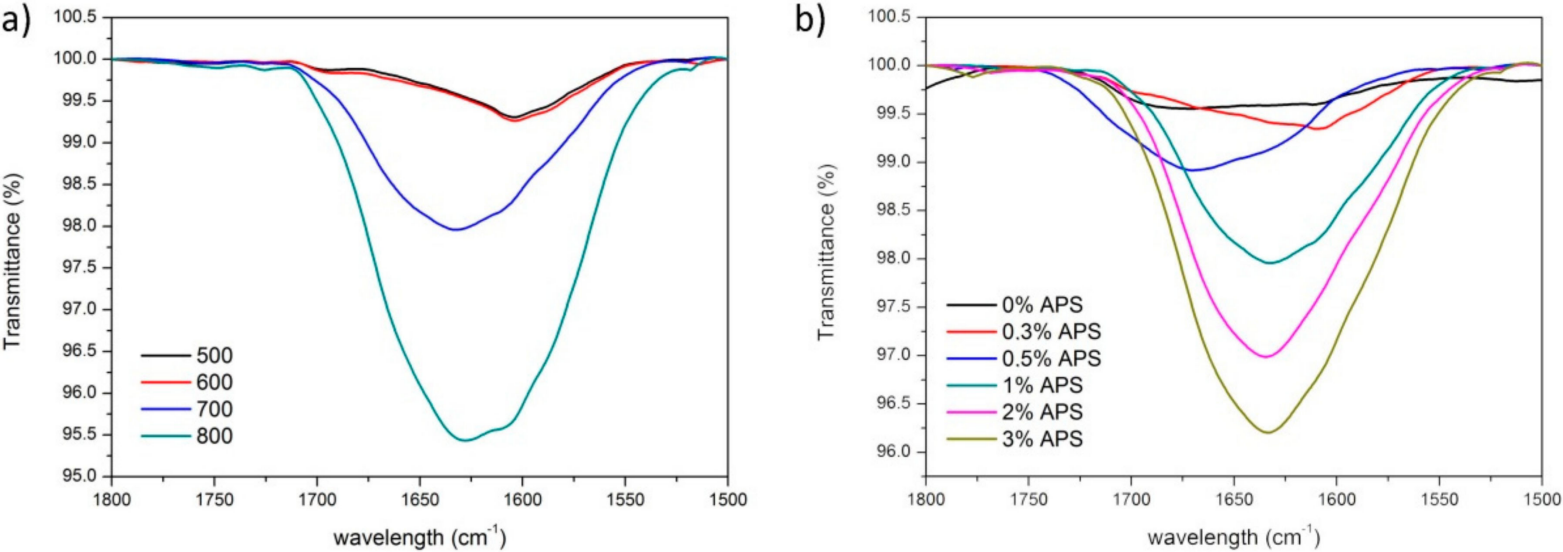
FTIR ATR spectra of the tenofovir (TFV) loaded particles (**a**) containing 1% silane in solution and pyrolyzed at different temperatures and (**b**) functionalized with different amounts of γ-APS and pyrolyzed at 700 °C.

Similarly, as we have performed in the previous section, we also determined the interactions occurring between the surface of the functionalized hybrid particles and the functional groups of the drug molecule. Tenofovir possesses a complex FTIR spectrum where various N–H wagging bands and various C–H out of plan deformation appear in the range of 900–600 cm^−1^. The C–N and C=N bonds are found around 1250 cm^−1^ for the medium deformation stretch of the single bond whereas the aromatic pairs appear at 1410 and 1450 cm^−1^. At 1695 cm^−1^ it is located the band corresponding to the stretching mode of the phosphate group and the medium stretch of the NH_2_-scissoring band at 1550 and 1570 cm^−1^ [39]. In the presence of water, the NH stretching vibrations usually present a red shift of about 10 cm^−1^ whilst the NH_2_ scissoring remain unaffected [40]. In the spectra presented in Figure 5 it is observed that the bands corresponding to the γ -APS functionalization partially overlap the bands corresponding to the TFV loaded on the particles. We cannot distinguish the effective contribution of neither the silane nor the tenofovir but, in general, as the pyrolysis temperature increases, and for the same amount of silane (Figure 2a), it is observed an increase in the band intensity and broadness, indicating that both components are adhered to the surface. Focusing our attention on just one pyrolysis temperature (Figure 2b), the general trend is an increase of the intensity with the γ-APS concentration. This increase could be, however, attributed to the contribution of the γ -APS bands to the spectra.

**Figure 6:**
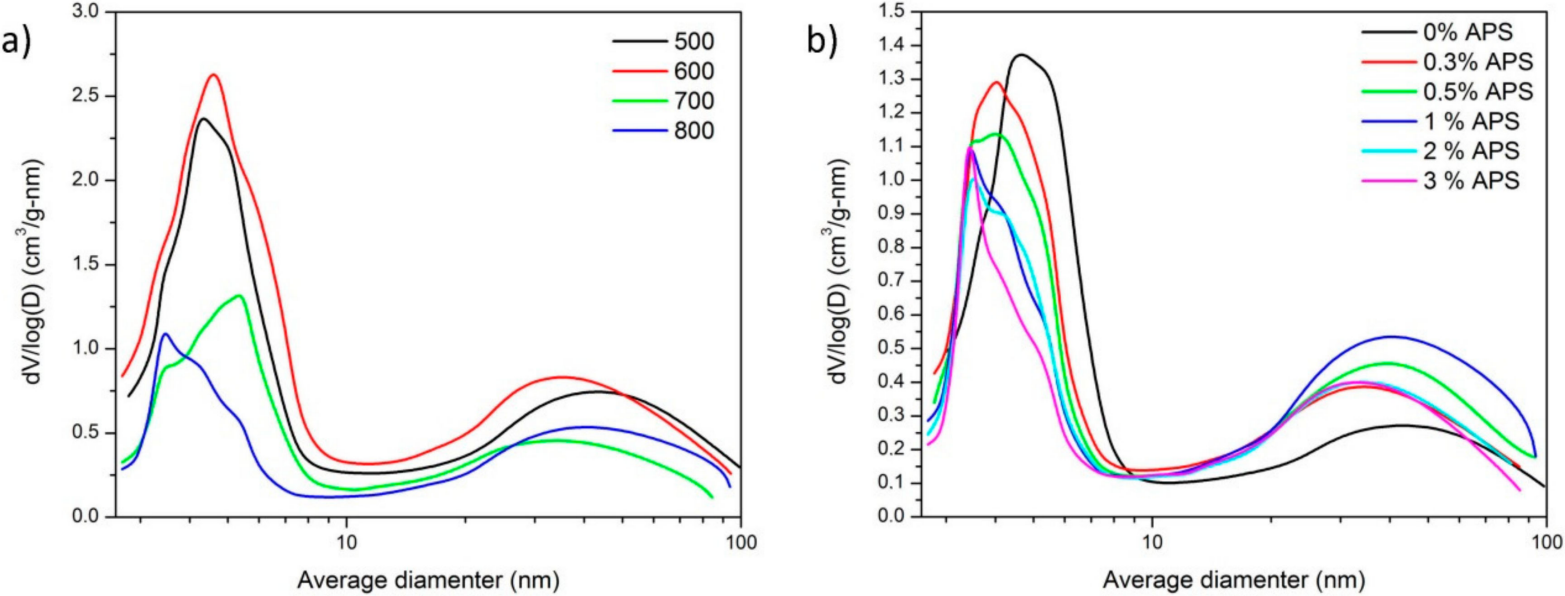
Pore Size Distributions of the TFV loaded particles (**a**) containing 1% silane in solution and pyrolyzed at different temperatures and (**b**) functionalized with different amounts of γ-APS and pyrolyzed at 800 °C.

The loaded TFV molecules induce a decrease of the SSA, especially in those samples pyrolyzed at 700 and 800 °C. Table 3 collects the determined BET SSA in the loaded samples as well as the micro and mesopore volume, as calculated through the application of the t-plot method and BJH methods, respectively. In the samples heat treated at 500 and 600 °C we just found slight variations in the mesopore volume whereas the volumes of the micropores remain almost unaltered. By increasing the temperature, the mean pore size, as calculated from the pore size distributions decreases progressively (Figure 6a), this decrease being more pronounced when the amount of γ-APS increases (Figure 6b).

### 3.4. Drug Releasing Capability and Pharmacokinetical Data

In order to examine the drug loading and releasing capacity, UV-Vis spectroscopy (spectra not shown here) was used to determine the concentration in solution of the drug TFV after being loaded in the particles. The time at which the maximum loading capacity is achieved was determined by collecting an aliquot of the loading liquor and measuring the amount of TFV in solution at the specific time (Supplementary Material Figure S5). All the experiments were carried out with the same amount of SFV, meaning that, after extracting the aliquot, the SVF was replaced with the same volume of fresh solution. In the samples pyrolyzed at low temperature, there is a maximum drug loaded capability which is acquired by immersing the materials into the TFV solutions for less than 30 s. Then, the amount of TFV in solution increases again since the affinity of the drug for the material is lower than for the H_2_O molecules. In the case of the samples heat treated at 700 and 800 °C, the maximum amount of drug loaded is acquired by maintaining the materials in solution for more than 180 s.

The mechanism of drug release can be fitted in accordance with various mathematical kinetic models. The description of these models can be found elsewhere [41]. The First Order kinetic model implies that the release rate is only dependent on the concentration of the drug in the releasing media and represents the drug release as a function of surface action. This kinetic model is usually applied to porous matrices.

(1) $$\frac{M_{t}}{M_{\infty }}=1-e^{-K_{1st}t}$$

where *M_t_* and *M_∞_* are the cumulative absolute amount of drug released at time t and at infinite time, respectively. M_∞_ can be taken as maximum drug released capability. Kinetic parameters obtained through the application of the First Order kinetic model are found in Table S1

The Hopfenberg release kinetics usually applies to surface-eroding dosage forms and in its expression (Equation (2)), *K_HF_* is the Hopfenberg rate constant, which includes the expression *K_0_/M_0_a_0_*, in which *K_0_* is the erosion rate constant, *M_0_* is the initial drug concentration in the dosage form and *a*_0_ is the radius of the sphere or cylinder or the average thickness of the slab (depending on formulation). The variable *n_H_* is the Hopfenberg exponent, which is related to the geometry (having a value of 1 for slabs and films, 2 for cylinders and 3 for spheres) [42].

(2) $$\frac{M_{t}}{M_{\infty }}=1-{\left(1-K_{HF}t\right)}^{n}$$

The application of the Hopfenberg release kinetics model allows the obtaining of the kinetic parameters collected in Table S2.

The Korsmeyer–Peppas kinetic model describes the drug release as a function of time and its general equation responds to the following expression:

(3) $$\frac{M_{t}}{M_{\infty }}=K_{KP}t^{n}$$

Here, the *n* is the exponent indicates the mechanism responsible for the drug release. When diffusion predominates, the value of *n* is less than or equal to 0.5 (in such a case, it will also fit to the Higuchi kinetic model). When the n value is between 0.5 and 1.0, it is an indicator of an “anomalous transport” based on diffusion and the structural modification of the dosage form. If *n* acquires a value equal to 1.0 (“transport case II”) and over 1.0 (“transport super-case II”) it is characteristic of drug releases that are due only to structural changes in the formulation. The kinetic parameters obtained by the application of this kinetic model is found in Table S3.

The Weibull kinetic model is an empirical equation that can be applied to almost all dissolution/release process of drug systems in different dosage forms:

(4) $$\frac{M_{t}}{M_{\infty }}=1-\frac{e^{-{\left(t-T_{1}\right)}^{b}}}{a}$$

In Equation (4), the parameter *T_1_* is the lag time, which is usually zero, a is a scale parameter that describes dependence on time and *b* takes different values as a function of the shape of the dissolution curves (*b* = 1 indicates exponential curve, *b* = 2 fits for sigmoidal curves and *b* = 3 indicates parabolic curves) [43]. The kinetic parameters obtained by the Weibull model are collected in Table S4.

**Figure 7:**
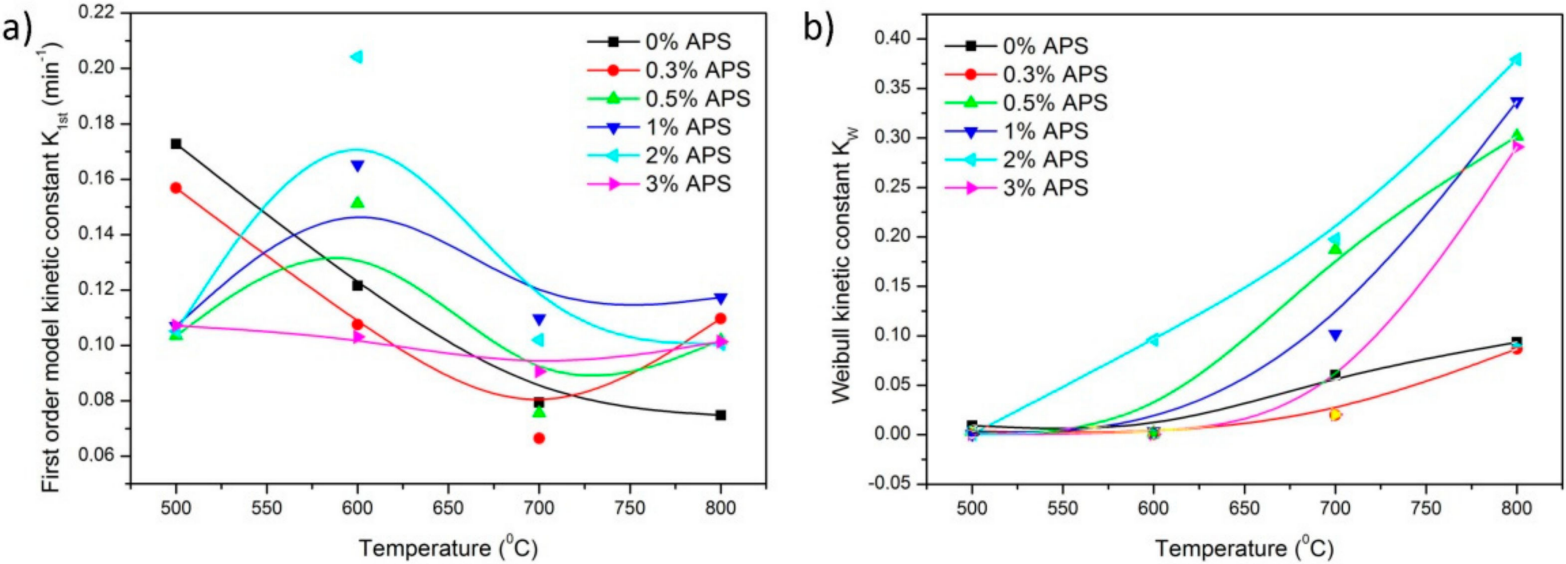
Kinetic constants obtained from the fittings of the release profiles to different kinetic models (**a**) First order kinetic model and (**b**) Weibull kinetic model. In the x-axis it is shown the temperature at which the particles were obtained.

Two different behaviors are found in the materials. When the materials are produced at low pyrolysis temperatures, the dissolution kinetics fits better for a first order kinetic model although it slightly deviates for high γ-APS concentration. When the pyrolysis temperature used for synthesizing the particles are of 700 °C and beyond, the first order kinetic model may be also used, but the Weibull model might also apply. The kinetic constant obtained from the fitting to the first order kinetic model is dependent on the functionalization of the hybrid particles, as shown in Figure 7.

We observe that with no functionalization and γ-APS concentrations of 0.3 and 3% (i.e., the minimum and maximum values studied here), the kinetic constant obtained from the application of the first order model (Figure 7a) decreases progressively with the temperature of the pyrolysis of the materials whereas, at medium γ-APS concentrations, it is observed a maximum in the release constant at pyrolysis temperature of 600 °C. The application of the Weibull model, however, throws a distinct behavior in the evolution with the pyrolysis temperature of the materials (Figure 7b). By the application of this model, the kinetic constant increases with the pyrolysis temperature, a trend which is more pronounced at intermediate functionalization degrees. The *b* values experiment includes a maximum at γ-APS concentration of 0.5% indicating a tendency of a sigmoidal behavior in the release profile rather than exponential.

**Figure 8:**
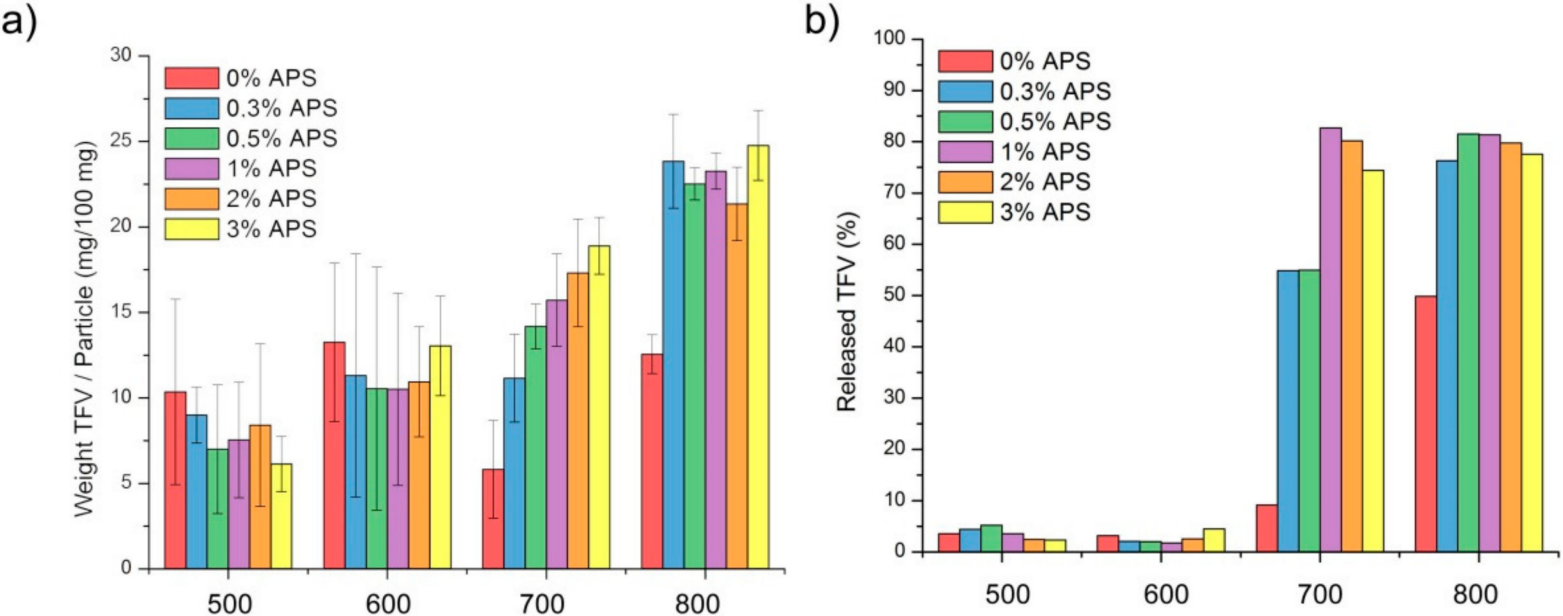
(**a**) mg of TFV adsorbed per 100 mg of particles (**b**) Percentage of TFV released after 180 min in SVF. In the x-axis it is shown the temperature at which the particles were obtained.

According to the above mentioned values and the maximum load capacity obtained from the fittings (*M_∞_*), Figure 8a, the sample presenting the maximum loading capacity, is the one pyrolyzed at 800 °C and the amount of surface functional groups does not exert any influence on their loading capacity. On contrary, the sample pyrolyzed at 700 °C present a clear dependence of the functionalization with the loaded amount of TFV. Notice that these graphs are not normalized to the SSA. The maximum amount of TFV adsorbed (Figure 8a) has been used to calculate the released percentage of TFV (Figure 8b). The loading capability varies from 1 mg TFV/100 mg particles up to 25 mg TFV/100 mg particles, depending of the functionalization and the pyrolysis temperature. Taking into account these data, the TFV release profiles can be found in the Supplementary Material Figure S6.

As mentioned above, the two samples which present excellent delivery capability, with releasing percentages close to 90% of the adsorbed TFV (Figure 8b), are the ones pyrolyzed at 700 and 800 °C. On the other hand, neither the sample pyrolyzed at 500 nor the one heat treated at 600 °C exhibit an acceptable behavior for this drug since, apart from the poor uptaking capability, just 5% of the adsorbed TFV was released.

## 4. Discussion

The prepared micro-mesoporous hybrid materials have shown that the surface characteristics as well are going to determine their behavior as a drug carrier. The disposition of the γ-APS molecules on the surface of the hybrid materials plays an important role on the type of interactions with the drug molecule [44]. When these hybrid materials are treated at increased pyrolysis temperatures, new silanol bonds start to appear in the surface of the particles. These silanol groups may exert a strong influence on the surface characteristics and therefore the observed cytotoxicity of the particles against the different cell lines. Only in the materials pyrolyzed at 800 °C, a cytotoxic effect was observed, especially noticeable in the MT-2 cell line. Some authors have already addressed the importance of the nature and accessibility of the silanol bonds on the toxic effects of the inorganic materials [45,46]. It has been also demonstrated that the sensitivity of each cell line is completely different and dependent on multiple variables in the materials. Cell lines that are sensitive to surface silanol bonds might not be affected by the surface charge or particle size [47]. The MT-2 lymphoblastic cell line, has demonstrated being the most sensitive to the surface stress induced by the surface silanol bonds followed by uterus/endometrium epithelial cell line, HEC-1-A. The higher susceptibility of the MT-2 cell line against the silanol groups has been also reported by other authors and attributed the toxicity of the silica nanoparticles in normal human fibroblast cells than in cancer cells to their metabolic activity [48].

**Figure 9:**
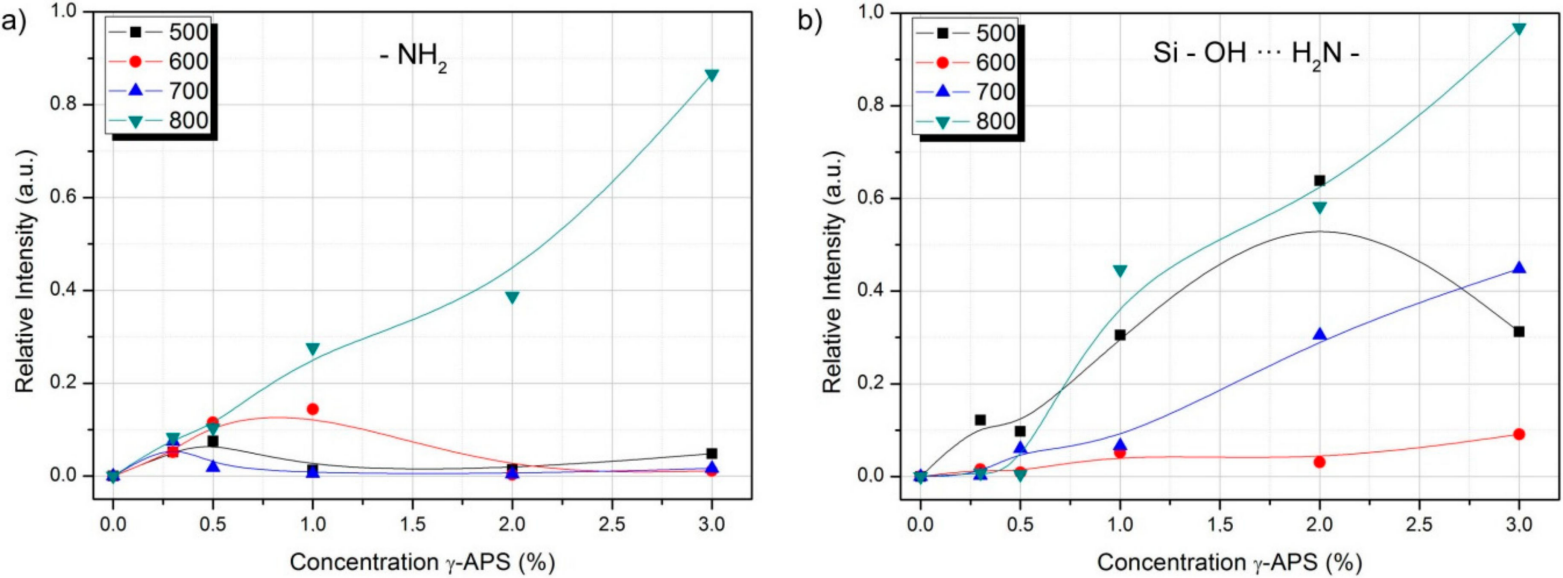
Relative intensity of the IR bands after spectral deconvolution. It is shown the relative intensity of the bands corresponding to (**a**) free amino groups and (**b**) amino groups interacting (weak interaction) with silanol groups.

Therefore, we have performed the deconvolution analysis of the FTIR spectra in the region where the principal bands of the silane coupling agent appears in order to determine the availability of the functional groups prone to interact with the TFV. As shown in Figure 9, the relative intensity of the band corresponding to free amino groups increases with the amount of γ-APS in solution only in the sample pyrolyzed at 800 °C. At lower temperature, there is a maximum at the concentrations 0.5%, 1% and 0.25% γ-APS for the samples pyrolyzed at 500, 600 and 700 °C, respectively. These differences are due to the different surface characteristics of the materials pyrolyzed at different temperatures. In the case of the materials pyrolyzed at low temperatures, the presence of organic residues coming from the PDMS minimized the adsorption of the silane coupling agent, creating a partially hydrophobic surface.

The grafted silane forms weak interactions the silanol groups close by either coming from the silanol functionalities of the surface or with some other hydrolyzed groups of the silane [49]. This is the case of the samples pyrolyzed at 500 and 600 °C, that forms γ-APS aggregates not attached to the surface of the hybrid material. In the samples pyrolyzed at 700 °C, after the formation of the monolayer at the minimum concentration of γ-APS, the coupling agent molecules start interacting. Solely in the case of the sample pyrolyzed at 800 °C, we can found the same proportion of free amino groups and zwitterionic forms.

These results can be further supported by the DSC analysis (Supplementary Material Figure S3). In the samples pyrolyzed at the lowest temperature, the γ-APS aggregates decompose at temperatures around 250 °C. By increasing the temperature to 600 °C, the interaction with the surface is slightly higher and the elimination of the adsorbed silane occurs exothermically at about 270 °C. In the sample pyrolyzed at 700 °C, the monolayer of γ-APS is eliminated through an endothermic reaction whereas the subsequent γ-APS layers formed afterwards are removed at temperatures close to 320 °C. Similarly to what happens in the sample heat treated at 800 °C, the decomposition temperature of the γ-APS decreases with the increased amount of γ-APS.

As multiple γ-APS layers are formed on the surface of the materials, the SSA decreases as well. The γ-APS is preferably linked on the surface of the larger pores, as deduced from the decrease of the mesopore volume in Table 1. The micropores are not accessible to the coupling agent in any case. The sample which possessed the highest surface area is the one pyrolyzed at 600 °C, nevertheless, because of the formation of the aggregates on the surface, we cannot appreciate a significant variation on the mesopore volume. After loading the TFV molecules, the SSA experiments a second decrease because of the filling of the small pores by the drug molecules. From the data collected in Table 1 and Table 3, we can appreciate a decrease of the SSA in all the materials loaded with TFV except in the samples pyrolyzed at 500 °C. This decrease is higher as the pyrolysis temperature increases and, again, is more pronounced in the sample pyrolyzed at 800 °C and increases with the functionalization. From Figure 8, one should expect a decrease in the SSA correlative with the amount of silane, since the sample pyrolyzed at 700 °C is the only sample where we did observe a relationship between the surface functionalization and the drug loading capacity.

Moreover, as we presented in Figure 6 there is a marked decrease of the mean pore size as the TFV enters into the porous particle structure. This decrease is more pronounced in the samples pyrolyzed at 700 and 800 °C and is somehow related with the γ-APS content in solution. In the PSD of the functionalized particles (not shown here) we did observe a decrease of the pore volume but not on the mean pore size whereas in the TFV loaded samples, the micro and mesopore volume is accompanied to a reduction in the mean pore diameter. This fact suggest that the γ-APS molecules fill the surface of the pores independently on their diameter, leading to a decrease in the pore volume but not in the mean pore size (as calculated from the PSD). The behavior of the TFV is completely different since the molecules occupy preferably the surface of the pores of about 8–9 nm whereas the small pores, of 3–4 nm are not reached by the drug.

As we mentioned above, there was an increase in the SSA in the sample pyrolyzed at the lowest temperature after the loading experiments. This increase of SSA could be attributed to the creation of new micropores (see Table 3) because of the stress created into the hybrid structure when the particles were immersed in the loading media. When the pyrolysis temperature increases, the hybrid structure possesses major consistency (i.e., becomes more inorganic) giving as a result a general decrease of the micropore volume because of the incorporation of the drug. Only in the sample pyrolyzed at 700 °C, where we encountered a direct relationship between the functionalization and the amount of drug loaded, both the micro and mesopore volumes decreased progressively with the functionalization. The presence of micropores or intrawall porosity exerts a hindrance in the diffusion of the drug molecules along the material [50]. The diffusion hindrance applies for both the loading and release capabilities, as already described in micro-mesoporous materials loaded with ampicillin [51] and dasatinib [52]. In these materials, the mechanism of TFV adsorption of the surface of the particles was described following the Langmuir model, which assumes the formation of a complete monolayer of the drug molecule on the surface of the porous particles. The surface energy was predicted to be homogeneous in such a way that all the surface sites present the same energy for adsorption [41]. Not only the presence of micro and mesopores but also the amino functionalization of the γ-APS influences TFV adsorption as the surface energy is modified, thus allowing a chemical interaction between the TFV molecules and the surfaces of the materials. Similar behavior has been described in the loading of different drug and organic molecules such molsidomin [53], methylene blue [54] or ibuprofen [55,56] on different mesoporous silicas with different crystallinity, morphology and surface areas. The surface amine functionalization turns out to be a determinant factor on the rate of adsorption. Moreover, the incorporation of lipid molecules, whose function is to act as the drug carrier in organic or hybrid hosts, may prevent the formation of strong covalent bonds between the functional groups of the matrix and the drug by creating hydrogen interactions with the participation of the amino groups of the lipid [57].

These results are in completely accordance with the observed drug uptake and release experiments in our functionalized hybrid particles. At the lowest pyrolysis temperatures, the drug uptake capability is the lowest (despite the high surface area found in the sample pyrolyzed at 600 °C) since there are almost no free –NH_2_ groups available to interact with the drug molecule. The release capability is also the lowest indicating that the interaction with the drug molecule is in form of a chemical bond. The releasing profiles can be adjusted to the first order kinetic model at low pyrolysis temperatures indicating that the tenofovir molecules are physiosorbed on the surface of the hybrid particles. At 700 and 800 °C, the release profiles are fitted better to the Weibull kinetic model, with a kinetic constant which is maximum at concentration of γ-APS 2%. At 700 °C we observe a strong correlation between the amount of γ-APS adsorbed on the surface and the capability of adsorbing TFV, whereas, in the case of the non-functionalized sample, the release capability is quite low, the incorporation of the γ-APS increases substantially the amount of TFV in the releasing media. It is noticed that, when the free –NH_2_ groups are present, the releasing capability is the lowest, a fact that is attributed again to the formation of the chemical bonds. The *b* values in the Weibull kinetic model are larger in the samples pyrolyzed at 700 °C than in the materials pyrolyzed at 800 °C indicating that the release of tenofovir occurs in a more sustained manner. We attribute this behavior to the presence of the silane monolayer attached to the surface of the materials that modulates the release of the drug molecule. Here, it should be clarified that these kinetic models are only valid for particles immersed in the medium and will be no longer applicable in the presence of a host matrix or any other surrounding medium which could somehow affect the diffusion and solubility of the drug molecules in the environment.

In the case of the sample pyrolyzed at 800 °C we reached releasing capabilities of about 95%. In this sample we have the same amount of free amino and zwitterionic forms, so the interaction of the drug molecule with the surface of the hybrid particles must be weak. The observed results are in agreement with the experiments reported by Lambert et al. [49] which observed the removal of attached biomolecules on functionalized silica because of the protonation of the zwitterionic bonds.

It is well known that ultrafine particles like silica, carbon black and TiO_2_ possess a high ability to induce inflammatory responses [58]. One strategy to minimize their toxicity is to encapsulate these particles into organic molecules thus conforming an organic-inorganic hybrid [59,60]. The toxicity experiments indicate that, the increase of the inorganic character and the presence of silanol groups of the sample pyrolyzed at 800 °C might exclude this sample in in-vivo experiments because of its interaction with the human cell lines. Similarly, in the samples pyrolyzed at 500 and 600 °C, we did observe some interactions and decay in the luminescent signal, especially at high cell concentrations. The incipient formation of a new carbon phase of small size [61] is the most probable cause of their cytotoxic behavior [62]. Although the most suitable technique to detect the carbonaceous phase appearing in this kind of materials is the Raman spectroscopy, the high fluorescent signal coming from the partially disintegrated hybrid organic-inorganic nanostructure, in many cases, hinders the D and G bands, typical of the carbon phase, and then the obtained results are not so reliable. Nevertheless, the indirect evidence of the presence of this carbon phase is the progressive change in the color of the samples which turns from white (in the as-prepared materials) to pale brown (in the sample pyrolyzed at 800 °C). When the pyrolysis temperature increases, so does the size of the carbon clusters and thus the particles are no longer cytotoxic.

The absence of any cytotoxic effect in the materials pyrolyzed at 700 °C, as well as the partial polymeric nature of the hybrid particles, makes these materials perfect candidates for their inclusion in polymeric vaginal gels. The drug adsorption capability can be also tuned depending on its degree of functionalization showing a linear response in both the adsorption characteristic as in the drug releasing capability with the amount of free amino groups in surface forming a monolayer. In our group, several mucoadhesive vaginal formulations have been already developed for the sustained release of TFV [22,24,25]. After the incorporation of the particles, the next step for the consecution of a ready-to use mucoadhesive vaginal formulation will be the determination of the behavior of the composite gel in in-vivo and in-silico experiments, as well as the possibility to manufacture these formulations in a cheap and fast manner for better accessibility to all the collectives potentially affected by VIH disease. In the foreseen future, our efforts will be clearly oriented to this direction.

## 5. Conclusions

New micro-mesoporous hybrid particles have been proved as successful hosts for the incorporation of TFV used for HIV prevention. The amount of drug loaded does not depend on the specific surface area but on the functionalization of the surface. At a certain pyrolysis temperature (700 °C), the surface of the particles is prone to interact with the silane coupling agent, thus forming a monolayer with free amino groups. These free amino groups allow the loading of a high amount of the drug TFV and the loaded amount depend on the functionalization. The kinetic profile in this material is sigmoidal rather than exponential because of the interaction between the amino functionalities and the drug molecule. At higher pyrolysis temperature, the amount of loaded drug is even higher, and so is the release capability, but it is independent on the amount of functionalization. This fact has been attributed to the presence of weak interactions (zwitterionic forms) between the amino functionalities. Apart from the direct relationship of the surface functionalization and the drug loading and release capabilities that presented the sample pyrolyzed at 700 °C, the absence of any cytotoxic effect in any of the cell lines tested, indicate that this material will be the perfect candidate to perform further in silico and in vitro experiments with the aim to develop a ready-to-use pharmacological formulation for HIV prevention. The actual solutions for the controlled release of TFV are directly related with the changes in the surrounding medium and the wettability of the host matrix but, for the first time; we report a material which provides a modulated response by direct functionalization. Thanks to the hybrid character of the particle, it is expected that the integration into a polymeric vaginal formulation occurs in a satisfactory manner. Nevertheless, in future, our efforts will be directed to the development of a manufacturable mucoadhesive vaginal formulation that include the developed hybrid particles specifically functionalized to achieve a sustained and modulate release of any antiviral molecule.

## Figures and Tables

**Table 1:** Specific surface area (as determined by BET), micropore (as determined by the t-plot method) and mesopore volume (determined from BJH method) of the prepared materials.

% APS	500			600			700			800		
	BET	V_mic_	V_meso_	BET	V_mic_	V_meso_	BET	V_mic_	V_meso_	BET	V_micr_	V_meso_
	(m^2^/g)	cm^3^/g		(m^2^/g)	cm^3^/g		(m^2^/g)	cm^3^/g		(m^2^/g)	cm^3^/g	
0	553	0.178	0.908	710	0.260	1.176	611	0.208	1.063	658	0.180	1.242
0.3	500	0.146	0.910	704	0.252	1.253	571	0.164	1.000	530	0.250	0.851
0.5	484	0.164	0.872	702	0.202	1.220	570	0.189	0.992	518	0.195	0.853
1	480	0.131	0.861	697	0.263	1.259	531	0.199	0.969	490	0.237	0.813
2	465	0.145	0.794	701	0.206	1.211	526	0.183	0.912	405	0.171	0.621
3	504	0.210	0.882	643	0.199	1.169	514	0.199	0.900	395	0.162	0.623

**Table 2:** Results of the cytotoxicity analysis of the materials tested in MT-2, THP-1 and HEC-1A cells. Cytotoxic Concentrations 50 (CC_50_) were calculated using GraphPad Prism software.

Pyrolysis Temperature (°C)	CC_50_ µg/mL (CI95%; R2)		
	HEC-1A	THP1	MT-2
500	>1000	>1000	>1000
600	>1000	>1000	>1000
700	>1000	>1000	>1000
800	≈1000	≈1000	447.5 (269.0–765.0; 0.9012)

**Table 3:** Specific surface area (as determined by BET), micropore (as determined by the t-plot method) and mesopore volumen (determined from BJH method) of the prepared materials loaded with the maximum amount of TFV.

% APS	500			600			700			800		
	BET	V_micro_	V_meso_	BET	V_micro_	V_meso_	BET	V_micro_	V_meso_	BET	V_micro_	V_meso_
	(m^2^/g)	cm^3^/g		(m^2^/g)	cm^3^/g		(m^2^/g)	cm^3^/g		(m^2^/g)	cm^3^/g	
0	534	0.119	1.456	679	0.158	1.348	562	0.270	1.133	427	0.139	0.600
0.3	554	0.003	1.131	687	0.235	1.402	551	0.129	1.126	316	0.131	0.607
0.5	594	0.028	1.233	669	0.173	1.407	459	0.082	0.985	281	0.109	0.615
1	546	0.056	1.147	665	0.226	1.376	398	0.071	0.844	243	0.094	0.614
2	517	0.255	1.059	653	0.189	1.354	325	0.047	0.707	230	0.083	0.521
3	537	0.186	1.100	626	0.178	1.365	323	0.082	0.751	202	0.015	0.499

## Supplementary Materials

The following are available online at https://www.mdpi.com/1996-1944/13/16/3494/s1, Figure S1: FTIR-ATR spectra in the spectral region 1400–1750 cm^−1^ for the samples pyrolyzed at (a) 500 °C, (b) 600 °C, (c) 700 °C and (d) 800 °C and functionalized with different amounts of γ-APS. In the legends, it is labeled the amount of -APS in solution (in %) followed with and N, Figure S1: Thermogrammes of the samples pyrolyzed at (a) 500 °C, (b) 600 °C, (c) 700 °C and (d) 800 °C and functionalized with different amounts of γ-APS, Figure S2: Differential Scanning Calorimetry curves of the samples pyrolyzed at (a) 500 °C, (b) 600 °C, (c) 700 °C and (d) 800 °C and functionalized with different amounts of γ-APS, Figure S3: Nitrogen adsorption and desorption isotherms of the samples pyrolyzed at (a) 500 °C, (b) 600 °C, (c) 700 °C and (d) 800 °C and functionalized with different amounts of γ-APS, Figure S4: Kinetic curves for the loading of tenofovir of samples pyrolyzed at (a) 500 °C, (b) 600 °C, (c) 700 °C and (d) 800 °C and functionalized with different amounts of γ–APS, Figure S6: Drug releasing curves of Tenofovir obtained in the samples pyrolyzed at (a) 500 °C, (b) 600 °C, (c) 700 °C and (d) 800 °C and functionalized with different amounts of γ-APS, Table S1: First order kinetic model, Table S2: Hopfenberg kinetic model, Table S3: Korsmeyer-Peppas kinetic model, Table S4: Weibull kinetic model.
