# Supplementary material for: Amino Functionalized Micro-Mesoporous Hybrid Particles for the Sustained Release of the Antiretroviral Drug Tenofovir
Source: Materials (Basel). 2020 Aug 7;13(16):3494. doi: 10.3390/ma13163494 (PMC7476006; doi:10.3390/ma13163494)
Supplement: Supplementary file 1 [file materials-13-03494-s001.pdf]

materials

Supplementary Materials

Amino Functionalized Micro-Mesoporous Hybrid Particles for the Sustained Release of the Antiretroviral Drug Tenofovir

Araceli Martín-Illana, Raul Cazorla-Luna, Fernando Notario-Pérez, Roberto Ruiz-Caro, Luis Miguel Bedoya, María Dolores Veiga-Ochoa, Juan Rubio and Aitana Tamayo

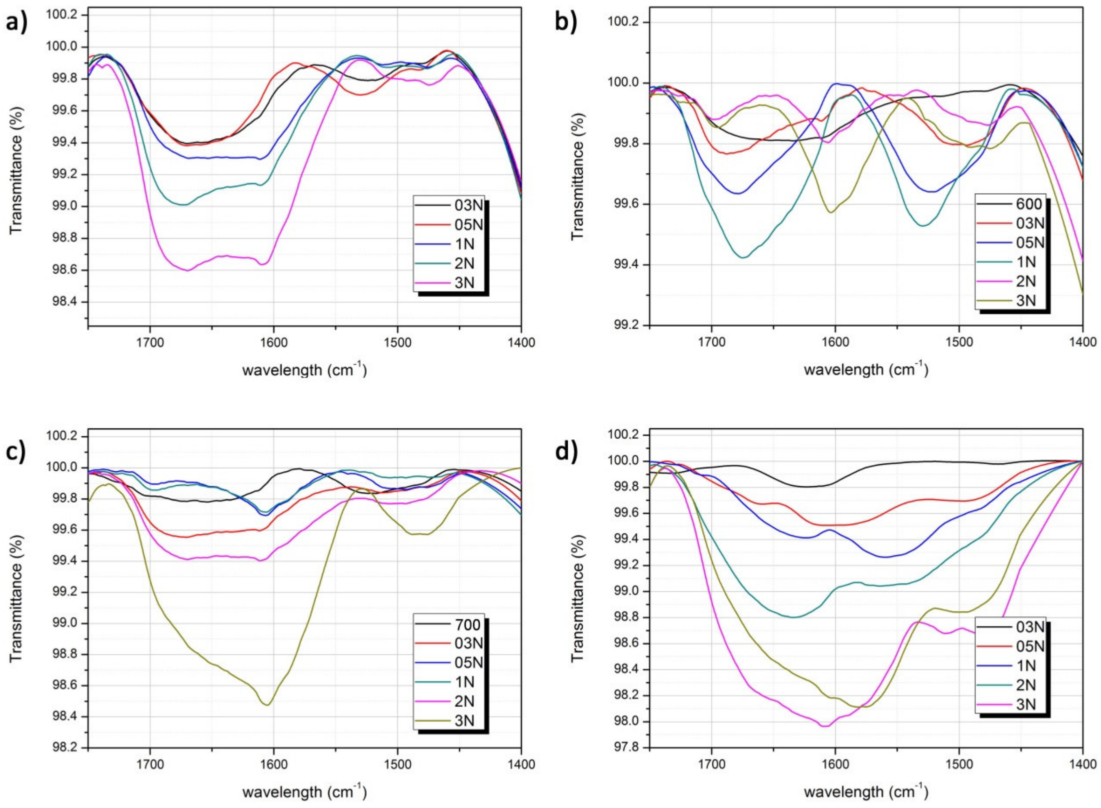

Figure S1. FTIR-ATR spectra in the spectral región 1400–1750 cm^{-1} for the samples pyrolyzed at (**a**) 500 °C, (**b**) 600 °C, (**c**) 700 °C and (**d**) 800 °C and functionalized with different amounts of -APS. In the legends, it is labeled the amount of γ-APS in solution (in %) followed with and N.

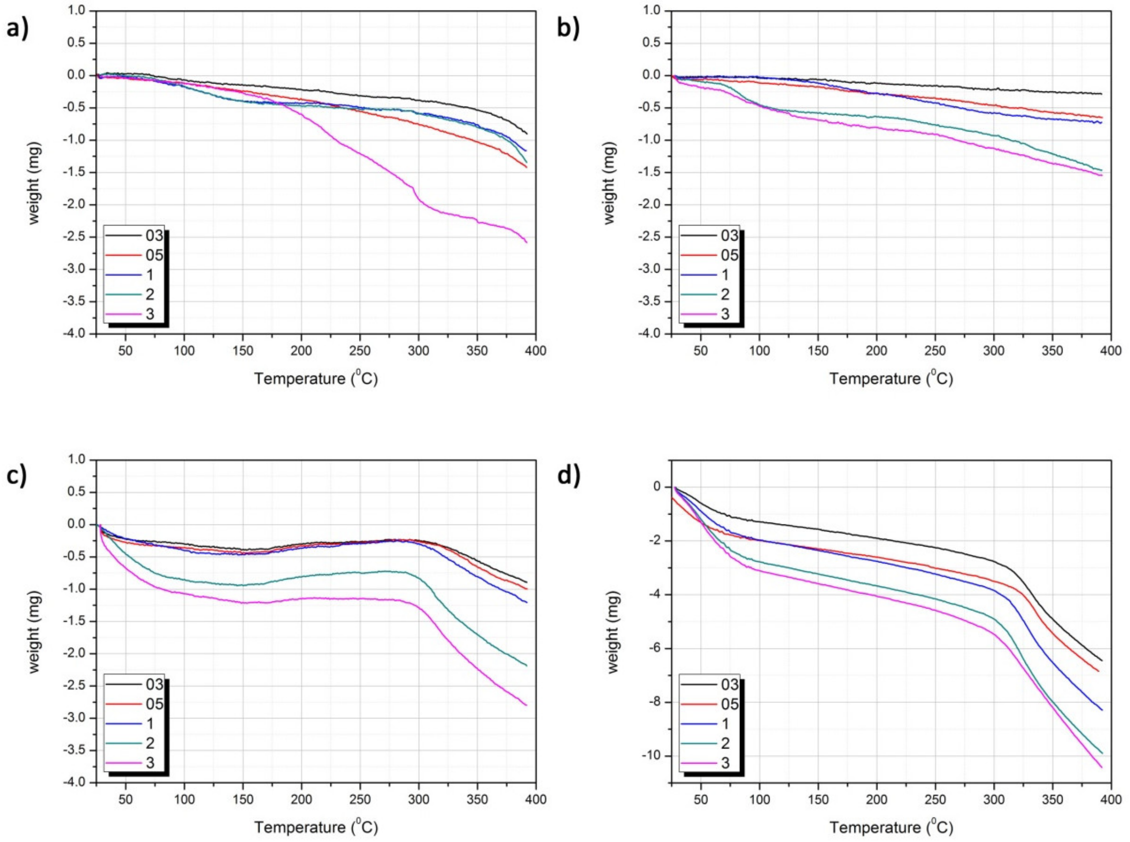

Figure S2. Thermogrammes of the samples pyrolyzed at (**a**) 500 °C, (**b**) 600 °C, (**c**) 700 °C and (**d**) 800 °C and functionalized with different amounts of -APS.

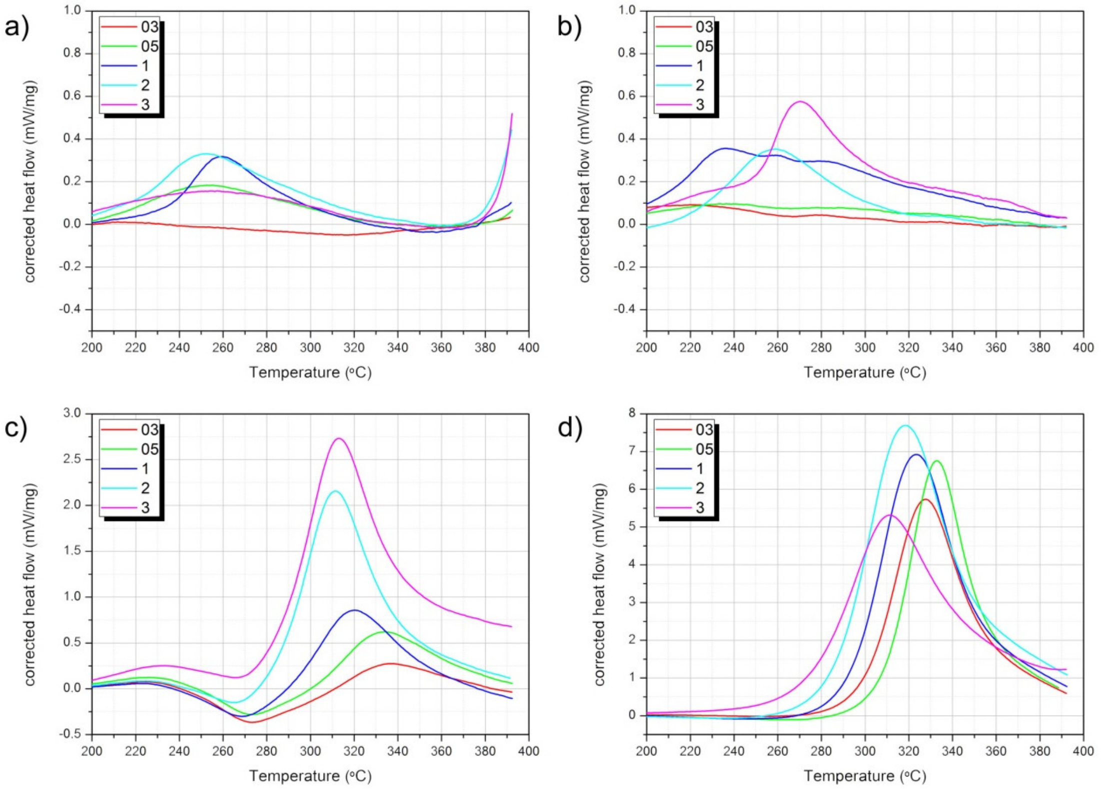

Figure S3. Differential Scanning Calorimetry curves of the samples pyrolyzed at (**a**) 500 °C, (**b**) 600 °C, (**c**) 700 °C and (**d**) 800 °C and functionalized with different amounts of -APS.

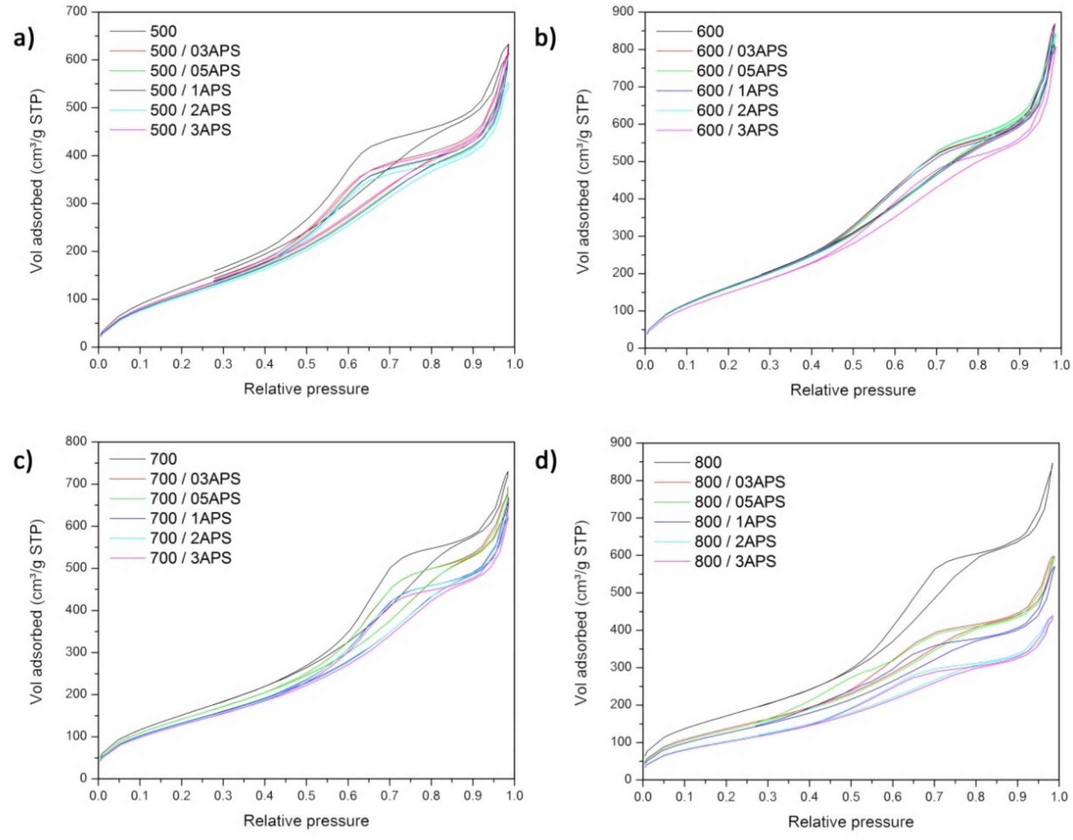

Figure S4. Nitrogen adsorption and desorption isotherms of the samples pyrolyzed at (**a**) 500 °C, (**b**) 600 °C, (**c**) 700 °C and (**d**) 800 °C and functionalized with different amounts of -APS.

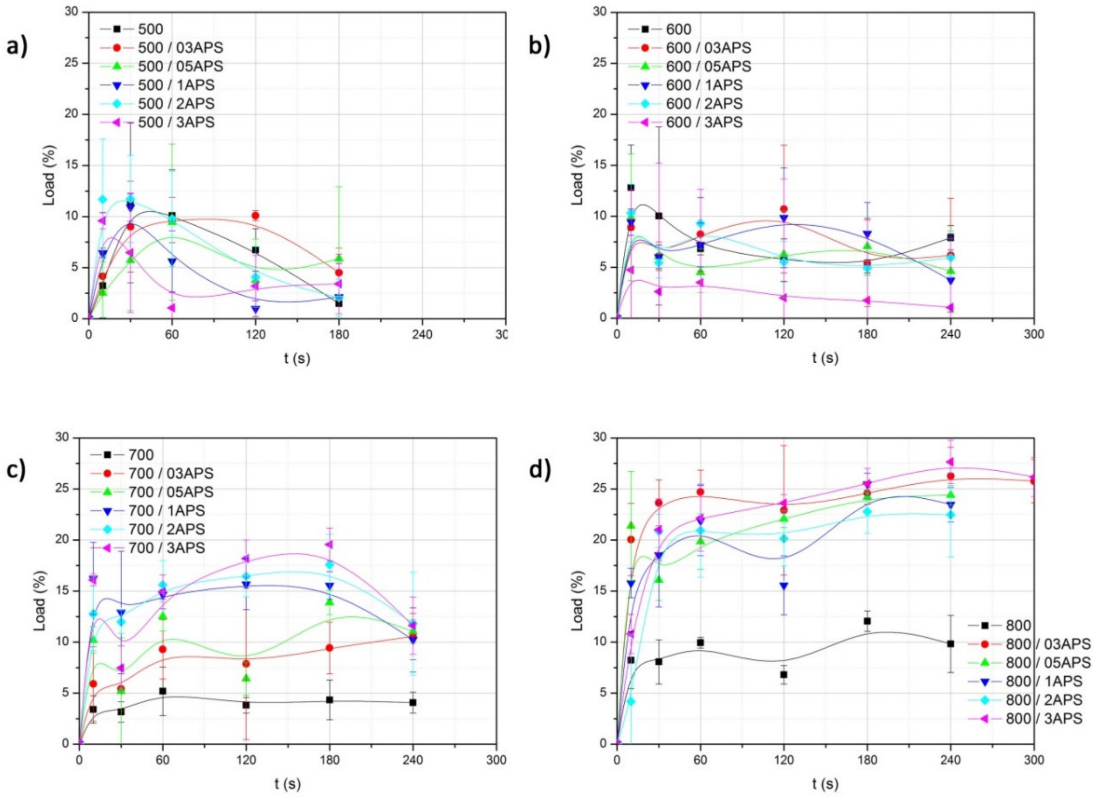

Figure S5. Kinetic curves for the loading of Tenofovir of samples pyrolyzed at (**a**) 500 °C, (**b**) 600 °C, (**c**) 700 °C and (**d**) 800 °C and functionalized with different amounts of ⊚-APS.

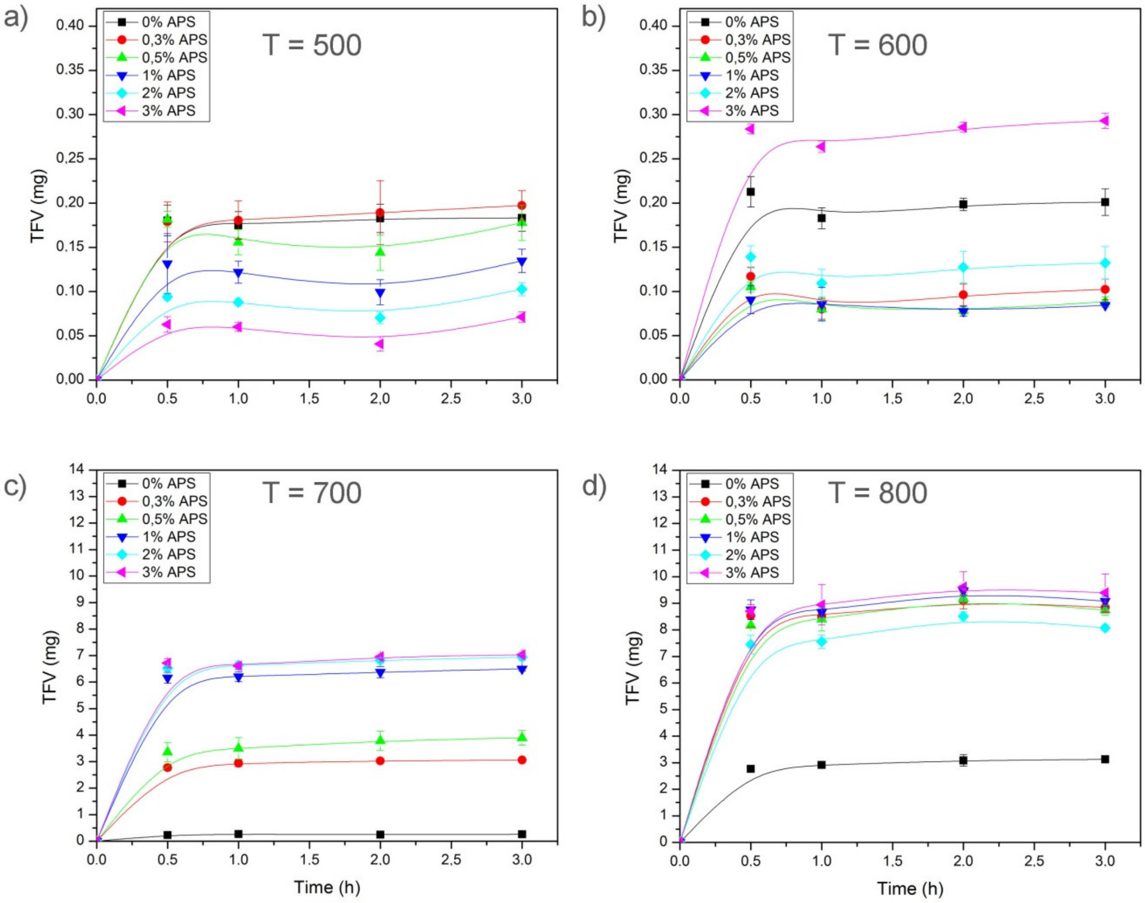

Figure S6. Drug releasing curves of Tenofovir obtained in the samples pyrolyzed at (**a**) 500 °C, (**b**) 600 °C, (**c**) 700 °C and (**d**) 800 °C and functionalized with different amounts of -APS

Kinetic parameters obtained from the fittings of the releasing curves of tenofovir to different kinetic models. (**Error! Reference source not found.**: First order kinetic model, **Error! Reference source not found.**: Hopfenberg kinetic model, **Error! Reference source not found.**: Korsmeyer-Peppas kinetic model: Weibull kinetic model, Table S1. Kinetic parameters obtained from the fittings of the releasing curves of tenofovir to the Weibull kinetic model.)

Table S1. parameters obtained from the fittings of the releasing curves of tenofovir to the first order kinetic model.

γ-APS	500				600				700				800			
	M_∞	K_{1st}	χ^2	r^2	M_∞	K_{1st}	χ^2	r^2	M_∞	K_{1st}	χ^2	r^2	M_∞	K_{1st}	χ^2	r^2
0	0.18	0.17	1.10E-05	0.998	0.20	0.12	1.64E-04	0.974	0.26	0.08	7.89E-05	0.992	3.06	0.07	4.56E-03	0.997
0.3	0.19	0.10	3.40E-05	0.994	0.10	0.11	2.35E-04	0.863	3.05	0.07	7.78E-03	0.995	8.84	0.11	4.21E-02	0.997
0.5	0.16	0.16	2.58E-04	0.944	0.09	0.15	6.82E-05	0.945	3.75	0.08	1.67E-02	0.992	8.75	0.10	8.20E-02	0.993
1	0.12	0.11	2.30E-04	0.910	0.08	0.17	2.37E-05	0.979	6.37	0.11	1.08E-02	0.998	9.06	0.12	8.12E-02	0.994
2	0.09	0.11	1.56E-04	0.886	0.13	0.20	1.24E-04	0.954	6.81	0.10	1.18E-02	0.998	8.02	0.10	1.22E-01	0.988
3	0.06	0.11	1.32E-04	0.797	0.28	0.10	1.62E-04	0.987	6.93	0.09	4.21E-02	0.994	9.29	0.10	6.28E-02	0.995

Table S2. Kinetic parameters obtained from the fittings of the releasing curves of tenofovir to the Hopfenberg kinetic model.

γ-APS	500					600					700					800				
	M_∞	K_{HP}	n	χ^2	r^2	M_∞	K_{HP}	n	χ^2	r^2	M_∞	K_{HP}	n	χ^2	r^2	M_∞	K_{HP}	n	χ^2	r^2
0	0.15	9.31	0.03	2.07E-05	0.996	0.19	0.02	0.05	3.89E-04	0.939	0.22	0.03	0.11	6.45E-04	0.937	2.71	0.02	0.14	4.74E-02	0.967
0.3	0.13	0.47	0.09	6.07E-05	0.989	0.11	0.00	0.07	3.99E-04	0.768	2.63	0.02	0.15	1.05E-01	0.927	8.59	0.01	0.08	2.37E-01	0.981
0.5	0.14	7.40	0.02	4.08E-04	0.911	0.09	0.00	0.01	1.17E-04	0.906	3.43	0.02	0.14	4.71E-02	0.978	8.36	0.02	0.09	1.98E-01	0.984
1	0.09	0.43	0.07	4.19E-04	0.835	0.08	0.23	0.01	4.69E-05	0.960	5.31	0.10	0.08	9.90E-02	0.985	9.39	0.01	0.07	1.91E-01	0.985
2	0.06	0.47	0.08	2.51E-04	0.818	0.13	0.00	0.01	1.72E-04	0.937	6.04	0.04	0.09	1.39E-01	0.981	8.47	0.01	0.10	1.66E-01	0.984
3	0.04	0.66	0.08	1.96E-04	0.699	0.30	0.01	0.09	4.49E-04	0.965	4.14	1.36	0.10	2.91E-01	0.961	8.90	0.02	0.09	1.84E-01	0.986

Table S3. Kinetic parameters obtained from the fittings of the releasing curves of tenofovir to the Korsmeyer - Peppas kinetic model.

γ-APS	500					600					700					800				
	M_∞	K_{KP}	n	χ^2	r^2	M_∞	K_{KP}	n	χ^2	r^2	M_∞	K_{KP}	n	χ^2	r^2	M_∞	K_{KP}	n	χ^2	r^2
0	0.16	0.99	0.03	2.07E-05	0.996	0.16	0.97	0.05	3.89E-04	0.939	0.21	0.70	0.11	6.45E-04	0.937	2.19	0.73	0.14	4.74E-02	0.967
0.3	0.14	0.91	0.09	6.07E-05	0.989	0.10	0.67	0.07	3.99E-04	0.901	2.09	0.70	0.15	1.05E-01	0.927	7.48	0.83	0.08	2.37E-01	0.981
0.5	0.15	0.99	0.02	4.08E-04	0.911	0.11	0.72	0.01	1.17E-04	0.906	2.77	0.69	0.14	4.71E-02	0.978	6.79	0.85	0.09	1.98E-01	0.984
1	0.12	0.77	0.07	4.19E-04	0.835	0.09	0.89	0.01	4.69E-05	0.960	5.17	0.86	0.08	9.90E-02	0.985	7.23	0.90	0.07	1.91E-01	0.985
2	0.08	0.78	0.08	2.51E-04	0.818	0.13	0.91	0.01	1.72E-04	0.937	5.64	0.81	0.09	1.39E-01	0.981	6.41	0.80	0.10	1.66E-01	0.984
3	0.05	0.90	0.08	1.96E-04	0.699	0.22	0.86	0.09	4.49E-04	0.965	5.47	0.78	0.10	2.91E-01	0.961	6.97	0.87	0.09	1.84E-01	0.986

Table S4. Kinetic parameters obtained from the fittings of the releasing curves of tenofovir to the Weibull kinetic model.

γ-APS	500					600					700					800				
	M_∞	b	a	χ^2	r^2	M_∞	b	a	χ^2	r^2	M_∞	b	a	χ^2	r^2	M_∞	b	a	χ^2	r^2
0	0.18	2.08	107	1.44E-05	0.997	0.20	2.70	903	1.51E-04	0.976	0.26	1.09	16.5	1.01E-04	0.990	3.07	0.92	10.7	5.77E-03	0.996
0.3	0.19	0.53	2.76	3.15E-05	0.994	0.10	2.98	2620	2.36E-04	0.863	3.01	1.43	50.6	2.35E-03	0.998	8.82	1.09	11.5	5.57E-02	0.995
0.5	0.16	2.48	388	3.22E-04	0.930	0.09	2.54	492	8.06E-05	0.935	3.84	0.67	5.4	1.42E-02	0.993	8.91	0.60	3.3	6.75E-02	0.994
1	0.12	2.85	1632	2.54E-04	0.900	0.08	2.41	293	2.86E-05	0.976	6.37	1.03	9.8	1.44E-02	0.998	9.18	0.61	3.0	9.60E-02	0.993
2	0.09	2.84	1563	1.86E-04	0.864	0.13	2.25	5	1.76E-04	0.936	6.81	1.02	10.4	1.57E-02	0.998	8.29	0.50	2.6	9.23E-02	0.991
3	0.06	2.84	1556	1.66E-04	0.746	0.28	2.79	1263	1.56E-04	0.988	6.86	1.54	48.9	3.35E-02	0.996	9.46	0.61	3.4	4.48E-02	0.997

1.

© 2020 by the authors. Submitted for possible open access publication under the terms and conditions of the Creative Commons Attribution (CC BY) license (http://creativecommons.org/licenses/by/4.0/).
